# LC–HRMS for the Identification of Quercetin and Its Derivatives in *Spiraea hypericifolia* (Rosaceae) and Anatomical Features of Its Leaves

**DOI:** 10.3390/plants12020381

**Published:** 2023-01-13

**Authors:** Natalia V. Petrova, Alexander A. Chernonosov, Vladimir V. Koval, Valeriya Yu. Andreeva, Andrey S. Erst, Alexander A. Kuznetsov, Maxim S. Kulikovskiy, Wei Wang, Sheng-Xiang Yu, Vera A. Kostikova

**Affiliations:** 1 Komarov Botanical Institute, Russian Academy of Sciences (BIN RAS), 197376 St. Petersburg, Russia; 2 Institute of Chemical Biology and Fundamental Medicine, Siberian Branch of Russian Academy of Sciences (ICBFM SB RAS), 630090 Novosibirsk, Russia; 3 Pharmaceutical Faculty, Siberian State Medical University, 634050 Tomsk, Russia; 4 Central Siberian Botanical Garden, Siberian Branch of Russian Academy of Sciences (CSBG SB RAS), 630090 Novosibirsk, Russia; 5 Laboratory Herbarium (TK), Tomsk State University, 634050 Tomsk, Russia; 6 K.A. Timiryazev Institute of Plant Physiology, Russian Academy of Sciences (IPP RAS), 127276 Moscow, Russia; 7 State Key Laboratory of Systematic and Evolutionary Botany, Institute of Botany, Chinese Academy of Sciences, Beijing 100093, China; 8 College of Life Sciences, University of Chinese Academy of Sciences, Beijing 100049, China

**Keywords:** *Spiraea hypericifolia*, Chamaedryon, quercetin, glycoside, anatomy

## Abstract

*Spiraea hypericifolia* L. is affiliated with the section *Chamaedryon* Ser. of the genus *Spiraea* L. (Rosaceae). Similar to many other *Spiraea* species, *S*. *hypericifolia* most often accumulates flavonols among other flavonoids, in particular quercetin and its derivatives. An ethanol–water extract from the aerial part of *S. hypericifolia* collected in the vicinity of the Ilyichovo settlement (Krasnoyarsk Krai, Russia) was analyzed by liquid chromatography with high-resolution mass spectrometry. Primary and secondary metabolites were found in the extract; structural interpretation consistent with quercetin and its derivatives was proposed for 10 of them. Major compounds were various glycosides of quercetin containing glucose (four compounds), galactose (one compound), xylose (two compounds), arabinose (one compound), or rutinose (one compound) as a carbohydrate residue. Isorhamnetin and 3-*O*-methylquercetin-3′-*O*-β-D-glucopyranoside were identified among methyl-containing compounds. The latter compound and reynoutrin, rhamnetin-3-*O*-β-D-xylopyranosyl-β-D-glucopyranoside, and quercetin-3-O-(6″-O-malonyl)-β-D-glucoside have not been previously found in *S. hypericifolia*. Data on the presence of quercetin and its derivatives in the extract of *S. hypericifolia* expand the understanding of the possible practical use of this plant. In addition, the microscopic features of *S. hypericifolia* leaves were studied. The diagnostic features of the leaf blade necessary for the *authentication* of raw materials were revealed: straight-walled epidermis cells, stomata located on both sides of the leaf blade (amphistomatic type), two types of trichomes, and wrinkled cuticula with nodi. The main anatomical diagnostic features of the leaves of *S. hypericifolia* were determined, which makes it possible to assess the authenticity of the raw material.

## 1. Introduction

Flavonoids are a large family of natural compounds that are characterized by various structures, high and diverse biological activities, and low toxicity [1]. The development of new medicinal formulations based on flavonoid-rich plants is hampered by insufficient knowledge about their chemical composition [2], especially the composition of wild species still not used by humans [3,4].

Quercetin, that is, 3,5,7,3′,4′-pentahydroxyflavone, is a flavonoid that belongs to the flavonol subclass and is of particular interest because it can be found in most of the studied plants [1]. Quercetin consists of three rings (A, C, and B) with a skeleton of diphenyl propane (C6-C3-C6). The A ring is synthesized through condensation of malonylcoenzyme A formed by glucose metabolism. Rings B and C also arise as a result of glucose metabolism in the shikimic acid pathway with the formation of cinnamic acid and its reduced product, coumaric acid. Quercetin contains five hydroxyl groups at positions C-3, C-5, C-7, C-3′, and C-4′.

Quercetin is a pharmacologically active compound because it has various biological effects. In most cases, researchers focus on its antioxidant potential [5,6,7,8]; however, in many other fields, clinical studies on quercetin have revealed that it is a potent pharmaceutical agent [2,9,10,11,12,13,14,15,16,17,18,19,20,21,22]. Quercetin has manifested its efficacy in a number of medical areas, such as allergology, immunology, endocrinology, gastroenterology, and urology, and is promising for application in psychiatry and oncology. Animal studies indicate that oral administration or inhalation of quercetin (20 mg/kg) has an antiasthmatic effect [9]; quercetin’s anti-inflammatory effect has been reported too [10,11]. Quercetin is beneficial in the treatment of a number of pelvic disorders (such as cystitis and chronic prostatitis) [12,13,14,15]. This compound is used for the treatment of cardiovascular diseases [16] and autoimmune disorders as well [17]. Recent studies suggest that quercetin exerts an antitumor effect as a human cathepsin B inhibitor [18] and suppresses the proliferation and metastatic spread of several cancer cell types, such as breast cancer [19], colon cancer [20], lung cancer [21], and pancreatic cancer cells [22].

*Spiraea* L. species are flavonoid-accumulating plants whose extracts may contain up to 120 mg/g flavonoids [23]. From data about phenolic compound evolution in the *Spiraea* genus, E.A. Karpova and N.P. Lapteva concluded that this genus’s flavonoid compounds are mainly represented by flavonols, with quercetin accounting for 50% to 90% of aglycons, and the remaining flavonoid compounds are represented by kaempferol and isorhamnetin and their derivatives [24]. A literature review of the chemical composition of *Spiraea* species also showed that the majority (over 25) of flavonoid compounds are quercetin and its derivatives [25]. During an investigation of the chemical composition of some *Spiraea* species, we attempted to study phenolic components of *Spiraea* representatives from Asian Russia, including *Spiraea hypericifolia* L. [26,27]. Data on other chemical components of *S. hypericifolia* L. are fragmentary; the plant has been found to contain such compounds as flavonols, proanthocyanidins, and catechins [28]. Quercetin and its derivatives in *S. hypericifolia* have not been studied separately.

*Spiraea hypericifolia* L. is affiliated with the section *Chamaedryon* Ser. of the genus *Spiraea* L. (Rosaceae). This plant is a shrub up to 80 (150) cm in height with brown smooth limbs (puberulent in young plants). It has long-ellipsoidal or lanceolar, grayish-green, smooth-margin leaves. Flowers are white and are bound in an attached umbrella blossom cluster; the fruit is a leaflet. *S. hypericifolia* occurs in Europe, the Caucasus, Central Asia, Russia, Mongolia, and China [29,30]. It grows on steppe slopes, meadows, and limestone outcrops [29].

The determination of the anatomical features of medicinal plants helps with quality control of the production of phytotherapeutics [31]. Anatomical and morphological features of medicinal plant raw materials are used as a mandatory indicator of standardization in regulatory documentation to confirm authenticity. Anatomical features are especially relevant for crushed raw materials and powders [32]. Falsification of drugs by replacement with a species of the same genus with similar pharmacological properties can be controlled through examination of diagnostic anatomical characteristics [33]. A comparative analysis of the stem and leaf structure of two closely related species, *Spiraea humilis* Pojark. and *Spiraea salicifolia* L., from Russia has revealed diagnostic features that can be employed for interspecies diagnosis for their practical medicinal use [34]. It is reported that stems of species of the *Spiraea* section can be diagnosed only according to the structure of the core and several quantitative features. Leaves of the species under study are diagnosed on the basis of thickness of the lamina near the midrib and in the areas distant from ribs, the thickness of the paxillate mesophyll, and the presence or absence of trichomes [34]. The anatomical structure of the *S. hypericifolia* leaf has not been studied in Russia.

This study is aimed at (i) assaying *S. hypericifolia* extracts for quercetin and its derivatives and (ii) anatomical and diagnostic leaf analysis.

## 2. Results and Discussion

### 2.1. Flavonoid Assay in an S. hypericifolia Extract by Liquid Chromatography Coupled with High-Resolution Mass Spectrometry (LC–HRMS)

The analysis of LC–HRMS data was performed to characterize primary and secondary metabolites, of which 10 were structurally interpreted as quercetin and its derivatives (Table 1).

Among substances identified in the water–ethanol extract of *S. hypericifolia*, only isorhamnetin is a methyl-containing derivative of quercetin, with the most substances detected being various quercetin glycosides (Table 1). 3-*O*-methylquercetin-3′-*O*-β-D-glucopyranoside contains both a methyl group (position C-3) and a carbohydrate residue (position C-3′). The carbohydrate part of the molecule is composed of mono- and disaccharides. Monosaccharides include pentoses (arabinose and xylose) and hexoses (galactose, rhamnose, and glucose), whereas disaccharides include rutinose. The carbohydrate moiety is usually attached at the C-3 position. In some cases (e.g., quercetin-3-*O*-(6″-*O*-malonyl)-β-D-glucoside), glucoside is additionally acylated with malonic acid on the sugar’s hydroxyl group.

A hypothesis that quercetin and its derivatives tend to accumulate not only in *S. hypericifolia* but also in other *Spiraea* species has been proved by other researchers. Literature data on the phytochemical content of certain species vary (data on some taxa are scarcer than fragmentary because they are reported in one article only); therefore, it is a foregone conclusion that the presence of the flavonoid family in *Spiraea* is diverse. As shown in Table 2, all the studied *Spiraea* species contain flavonols. We suppose that in terms of the general diversity of flavonols contained in *Spiraea*, attention should be drawn to *S. salicifolia*. Literature data on this species are more or less representative (34 flavonols) because this taxon has been a subject of phytochemical studies more often than others. It is difficult to assess the diversity of flavonols in *Spiraea* by means of studies on such phytochemically underinvestigated species as *S. albiflora*, *S. brahuica*, or *S. nipponica*, in which only one flavonol has been found.

Other researchers have reported that rutin, quercetin, avicularin, hyperoside, and isoquercitrin (identified by us) are present in *S*. *hypericifolia*, whereas quercetin-3-O-(6″-O-malonyl)-β-D-glucoside, reynoutrin, rhamnetin-3-*O*-β-D-xylopyranosyl-β-D-glucopyranoside, and 3-*O*-methylquercetin-3′-*O*-β-D-glucopyranoside have not been found previously in either this species or other *Spiraea* taxa.

Regarding the set of flavonoid compounds in *Spiraea* species, quercetin has proved to be the most common substance (in 21 species) in various studies; hyperoside has been detected in 14 species; isoquercitrin has been identified in 10 *Spiraea* species; and avicularin, rutin, and isorhamnetin have been found in 10 species by various authors [25].

Quercetin’s pharmaceutical characteristics have been investigated the best to date. Most of the studies deal with its aglycone form; however, plasma analysis performed after administration of quercetin shows that quercetin glycosides are the main circulating compounds [35]. Various derivatives of quercetin (e.g., glycosides and methyl esters) are mostly found in plants [36]. Frequent identification of aglycone in plant extracts is traditionally attributed to the fact that pure quercetin as a marker is more available commercially than its glycosides [37].

Quercetin glycosides arise via attachment of a sugar to quercetin by replacement of one of the hydroxyl groups, with subsequent formation of a glycoside linkage. Quercetin glycosylation may theoretically take place on any hydroxyl group, whereas most common quercetin glycosides have a sugar group at the C-3 position. The isoquercetin structure has been found to contain glucose attached to the OH group of quercetin at the C-3 position. The addition of galactose to the quercetin molecule at the same C-3 position leads to hyperoside, and the addition of a rhamnosyl group initiates the synthesis of quercitrin. Several quercetin derivatives contain disaccharides, such as rutinose or arabinofuranose; their attachment at position C-3 causes the formation of important compounds: rutin and avicularin, respectively [2].

There are also some methylated derivatives of quercetin. For instance, rhamnazin contains two methyl groups at positions C-7 and C-3′. Isorhamnetin is another methylated flavonol (C-3′ position), which can be glycosylated, giving rise to quercetin 3-*O*-rutinoside (narcissin) and other compounds.

A carbohydrate molecule attached to quercetin aglycone has been shown to improve water solubility, absorption, and other properties [38]. The examples are an enzymatically modified isoquercitrin and oligoglycosylated rutin. The former contains up to 10 glucose residues attached to the C-3 position, whereas the latter carries up to five additional glucose residues attached to rutin’s glucose residue. These compounds are dissoluble in water and are taken up by the human body better than other quercetin glycosides used as food additives in the USA and Japan [39].

Rutin’s pharmaceutical characteristics are studied best among all quercetin glycosides: this compound promotes mammalian smooth muscle relaxation [40], and rutin’s antioxidant effect protects hepatic cells [41], inhibits hemoglobin oxidation [42], and exerts an anti-inflammatory action [43,44]. Large reviews regularly mention the biological activity and therapeutic features of such quercetin glycosides as quercitrin [45], isoquercitrin [46], hyperoside [47], and reynoutrin [48]; some authors focus on the pharmacological activity of its methyl-containing derivative, isorhamnetin [49]. Nonetheless, quercetin derivatives that are less common in plants have attracted much attention. For instance, quercetin-3-O-(6″-O-malonyl)-β-D-glucoside has previously been found only in *Moringa oleifera* Lam. (Moringaceae) [50], *Morus alba* L. (Moraceae) [51], *Apocynum venetum* L. [52], and *A*. *hendersonii* Hook. f. (Apocynaceae) [53]. The therapeutic potential of this compound and that of rhamnetin-3-*O*-β-D-xylopyranosyl-β-D-glucopyranoside and 3-*O*-methylquercetin-3′-*O*-β-D-glucopyranoside are yet to be researched.

### 2.2. Anatomical Features of S. hypericifolia Leaves

**Figure 1:**
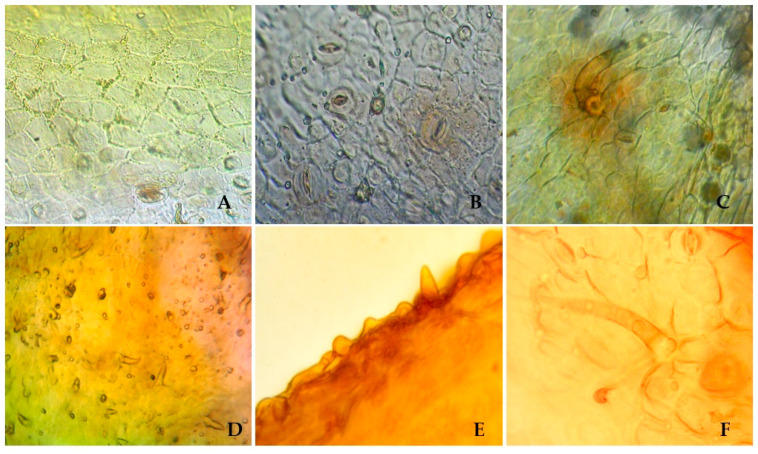
*S. hypericifolia* leaves. (**A**). The upper epidermis of the leaf blade (×400) (**B**). The lower epidermis of the leaf blade (×400) (**C**). The epidermis of the leaf blade with a single one-celled trichome and anomocytic stomata (×600). (**D**). Ordinary one-celled sharp conical trichomes (×600). (**E**). Wrinkled cuticula with nodi (×600). (**F**). An ordinary hair and its attachment site (×600).

To identify the species, the microscopic features of *S. hypericifolia* leaves were investigated next. Visual examination revealed straight-walled epidermis cells and anomocytic stomata located on both sides of the leaf, being numerous under the leaf (amphistomatic type) (Figure 1). Two types of trichomes can be found on both sides of the leaf: small ordinary one-celled sharp conical trichomes and larger ordinary one-celled thick-walled trichomes. The epiderma is covered with wrinkled cuticula with nodi. Calcium oxalate clusters can be seen in the leaf mesophyll.

There is not much information about the anatomical features of the leaves of *S. hypericifolia* in the literature. S. Kuzieva et al. [54] researched the structural features of the vegetative organs of *S. hypericifolia* growing in Uzbekistan. As a result, it has been shown that the leaves of this plant are amphistomatic; the epidermis consists of one row of cells with a thick-walled cuticle layer, and the stomata are not submerged. Leaf trichomes are also described, and according to these data, they are all of the same type: simple, opaque, awl-pointed, very rarely located along large veins. The discrepancy between the literature data and our findings may be explained by differences in the methods for obtaining micropreparations because those authors did not conduct special analyses of trichomes and merely recorded their presence on transverse sections of leaves near large veins. Additional research on this issue is required. Furthermore, for a comparative analysis in the future, it is important to study the anatomical and diagnostic features of other species of *Spiraea*, especially those with morphological features similar to those of *S. hypericifolia*.

## 3. Materials and Methods

### 3.1. Plant Material and Preparation of the Extract

Aerial parts of *S. hypericifolia* were collected during the flowering period in the vicinity of the Ilyichovo settlement, Shushensky District, Krasnoyarsk Krai (Russia). Voucher specimens (No. SH-KI-25, No. SH-KI-26, and No. SH-KI-27) were deposited in the plant material storage room in the Laboratory of Phytochemistry, CSBG SB RAS (Novosibirsk, Russia). Air-dried plant material was mechanically ground to obtain a homogeneous powder with a particle size of 2–3 mm. The dry extract was prepared as follows: The plant material was extracted in a water bath in three replicates (100 mL in the first replicate and 75 mL in the second and third replicates) with 70% ethyl alcohol for 8 h at 60 °C. After cooling, the combined filtrates were concentrated in a rotary evaporator to remove the solvent, and then the thick extract was dried in a vacuum drying cabinet to 5% residual moisture. To identify flavonoids, a stock solution of the crude extract was prepared by dissolving the dry extract in 70% ethanol at a 1:1000 ratio.

### 3.2. Mass Spectrometry Settings and the Spectral Library

LC–HRMS was carried out at the Core Facility of Mass Spectrometric Analysis at the Institute of Chemical Biology and Fundamental Medicine SB RAS (Novosibirsk, Russia).

An UltiMate 3000 liquid chromatograph (Thermo Fisher Scientific, San Jose, CA, USA) coupled with a Q Exactive HF mass spectrometer (Thermo Fisher Scientific) was utilized to determine the flavonoid profile of the *S. hypericifolia* extract. The chromatographic separation was attained at a 0.4 mL/min flow rate on a Zorbax Eclipse XDB-C18 reversed-phase column (150 × 3.0 mm, 5 μm, Agilent Technologies, Santa Clara, CA, USA) thermostatted at 40 °C. The mobile phase was composed of 0.1% aqueous formic acid (eluent A) and acetonitrile (eluent B). The elution gradient was implemented as follows: from 5% to 70% B for 40 min, followed by an increase to 90% B for 8 min, a decrease to 5% B for 5 min, and re-equilibration under the initial conditions for 7 min.

The parameters set for the electrospray ionization (ESI) source were as follows: electrospray voltage: 3.2 kV in the negative mode and 4.2 kV in the positive mode; capillary temperature: 320 °C; and S lens RF level: 50. Data were obtained by full-scan data-dependent acquisition (FS-dd-MS2) in the positive and negative modes at a resolving power of 45,000 full width at half maximum (FWHM) *m/z* 200. The following settings of the mass spectrometer were employed: scan range, *m/z* 80–1200; automatic gain control (AGC), 3e6; injection time, 100 ms; and isolation window, *m/z* 2.0. The normalized collision energy for the fragmentation of molecular ions was set to 40 eV. Targeted tandem mass spectrometry (MS/MS; dd-MS2) was performed in both positive and negative modes at 15,000 FWHM (*m*/*z* 200). AGC for dd-MS2 was set to 1e5, with an injection time of 50 ms and a loop count of 5. In the section of dd settings, the AGC target was programmed at 8e3, and the maximum injection time was set to 100 ms. Data were analyzed using Xcalibur 4.0 and the Compound Discoverer 3.1 software (Thermo Fisher Scientific). All the samples, including blank ones, were assayed in triplicate. All the samples were processed in Compound Discoverer 3.1 via a common workflow, “Environmental Unknown ID w Online and Local Database Searches” (Figure S1). A mass tolerance of 5 ppm was applied to all nodes. Several databases, namely, KEGG (https://www.genome.jp/kegg/, last accessed 10 March 2021), MassBank (https://massbank.eu/MassBank/, last accessed 10 March 2021), PlantCyc (https://plantcyc.org/, last accessed 10 March 2021), and Planta Piloto de Quimica Fina Universidad de Alcala (http://www.cqab.eu/index.php/en/, last accessed 10 March 2021), were chosen in ChemSpider.

Flavonoids were identified on the basis of both accurate mass and fragment mass “fingerprint” spectra via searches against the spectra of compounds available in the mzCloud database (https://www.mzcloud.org, last accessed 10 March 2021). If compounds were absent in mzCloud, they were tentatively identified using a ChemSpider search. According to the workflow, the masses extracted from the chromatograms were aligned and filtered to remove (i) background compounds present in the blank sample, (ii) substances that failed to become fragmented, (iii) compound masses that were absent in the databases, and (iv) signals with low intensity.

The *S. hypericifolia* extract and a blank sample, which consisted of pure solvent, were analyzed as two biological replicates with three technical replicates per treatment group.

### 3.3. Chemicals

All chemicals were of mass spectrometric or analytical grade. Chemical reference standards of quercetin and isoquercitrin were purchased from Sigma-Aldrich (Germany), whereas rutin, avicularin, and hyperoside from Fluka Chemie AG (Switzerland).

### 3.4. Anatomic Examination of S. hypericifolia Leaves

Leaves from the herbarium were studied visually with the naked eye using a 10× magnifier in accordance with the requirements of the books Herbae and Technique of Microscopic and Microchemical Studies of Herbal Drugs and Herbal Medicinal Products, State Pharmacopoeia of the Russian Federation, XIV edition [32].

Analyzed specimens were clarified by boiling in 5% sodium hydroxide and chloral hydrate. Micropreparations were examined under a BIOSCOP-1 biological microscope with 4×, 10×, and 40× lenses and 7×, 10×, and 15× oculars. Microphotoshooting was performed using a ToupCam FMA050 digital camera (12 megapixels).

## 4. Conclusions

Primary and secondary metabolites were detected in an ethanol–water extract of *S. hypericifolia*; 10 of these were identified as quercetin and its derivatives. Seven of the identified substances (quercetin, hyperoside, isoquercitrin, reynoutrin, avicularin, rutin, and isorhamnetin) have pharmacological activities, according to numerous studies. Hence, the therapeutic potential of *S. hypericifolia* has not been exploited so far. The pharmacological activity of other found substances (quercetin-3-O-(6″-O-malonyl)-β-D-glucoside, rhamnetin-3-*O*-β-D-xylopyranosyl-β-D-glucopyranoside, and 3-*O*-methylquercetin-3′-*O*-β-D-glucopyranoside) requires further investigation. Our data should improve the understanding of the enormous pharmacological potential of *S. hypericifolia*. Results on the content of quercetin and its derivatives (except for pharmaceutical compounds) may be of interest for food and cosmetics industries. The anatomical features of *S. hypericifolia* leaves were studied too. The anatomic diagnostic features of the raw material can be defined as straight-walled epidermis cells, stomata located on both sides of the leaf blade (amphistomatic type), two types of trichomes, and wrinkled cuticula with nodi. The anatomical features of *S. hypericifolia* leaves will allow investigators to evaluate the authenticity of raw materials, which have diverse uses.

## Figures and Tables

**Table 1:** Quercetin and its derivatives identified in water–ethanol extracts of *S. hypericifolia* by LC–HRMS using the databases mzCloud and ChemSpider.

ID	Identified Compounds	t_R_ (min)	Mode	Calculated Mass	Measured Mass	Delta Mass (Da)	Delta Mass (ppm)	MzCloud Score
1	Rutin *	9.22	Negative	610.15368	610.15338	0.00029	0.48	–
2	Hyperoside * (quercetin-3-galactoside)	12.39	Positive	464.09520	464.09548	−0.00027	−0.59	99.6
3	Quercetin *	12.40	Positive	302.04243	302.04265	−0.00022	−0.73	99.9
4	Rhamnetin-3-O-β-D-xylopyranosyl-β-D-glucopyranoside	12.48	Positive	610.15339	610.15338	0.00001	0.01	96.9
5	Isoquercitrin * (quercetin-3-O-β-D-glucopyranoside)	12.61	Negative	464.09548	464.09548	0.00000	0.00	99.4
6	Quercetin-3-O-(6″-O-malonyl)- β-D-glucoside	13.58	Positive	550.09574	550.09587	−0.00013	−0.24	97.5
7	Reynoutrin (quercetin-3-O-β-D-xylopyranoside)	13.72	Positive	434.08471	434.08491	−0.00020	−0.45	98.4
8	3-O-methylquercetin-3′-O-β-D-glucopyranoside	14.10	Positive	478.11095	478.11113	−0.00018	−0.37	97.6
9	Isorhamnetin (3′-methylquercetin)	14.10	Positive	316.05826	316.05830	−0.00004	−0.13	99.0
10	Avicularin * (quercetin-3-O-α-L-arabinopyranoside)	14.25	Negative	434.08417	434.08491	−0.00074	−1.71	–

Note: * Compounds confirmed by means of standards; “–”: only ChemSpider.

**Table 2:** Flavonoid distribution in *Spiraea* species (according to Kostikova and Petrova [25]).

Species	Numbers of Identified Flavonoids by Class						Total
	Flavones	Flavonols	Flavanones	Isoflavones	Catechins	Anthocyanins	
*S. aemiliana*	–	7	–	–	–	–	7
*S. albiflora*	–	1	–	–	–	–	1
*S. aquilegifolia*	–	4	–	–	–	–	4
*S. beauverdiana*	–	7	–	–	–	–	7
*S. betulifolia*	–	8	–	–	–	–	8
*S. brahuica*	2	1	–	–	–	–	3
*S. bumalda*	–	4	–	–	–	–	4
*S. canescens*	1	2	–	–	–	–	3
*S. cantoniensis*	–	3	–	–	–	–	3
*S. chamaedryfolia*	–	4	–	–	–	–	4
*S. crenata*	–	6	–	–	–	–	6
*S. dahurica*	–	2	–	–	–	–	2
*S. douglasii*	–	2	–	–	–	–	2
*S. elegans*	–	2	–	–	–	–	2
*S. flexuosa*	–	3	–	–	–	–	3
*S. formosana*	–	5	–	–	–	–	5
*S. humilis*	–	2	–	–	–	–	2
*S. hypericifolia*	4	6	–	–	6	–	16
*S. media*	–	9	–	–	–	–	9
*S. nipponica*	1	1	–	1	2	–	5
*S. prunifolia*	–	8	–	–	3	–	11
*S. pubescens*	–	2	–	–	–	–	2
*S. salicifolia*	4	34	–	–	3	–	41
*S. schlothauerae*	–	3	–	–	–	–	3
*S. sericea*	–	3	–	–	–	–	3
*S. trilobata*	–	4	–	–	–	–	4
*S. ussuriensis*	–	3	–	–	–	–	3

Note. “–“: data are unavailable.

## Data Availability

Raw data are available upon request.

## Supplementary Materials

The following is available online at https://www.mdpi.com/article/10.3390/plants12020381/s1, Figure S1: Workflow on Compound Discoverer used for flavonoid identification.
