# Supplementary material for: LC–HRMS for the Identification of Quercetin and Its Derivatives in Spiraea hypericifolia (Rosaceae) and Anatomical Features of Its Leaves
Source: Plants (Basel). 2023 Jan 13;12(2):381. doi: 10.3390/plants12020381 (PMC9861494; doi:10.3390/plants12020381)
Supplement: Supplementary file 1 [file plants-12-00381-s001.zip › plants-2135559-supplementary.pdf]

Supplementary Material

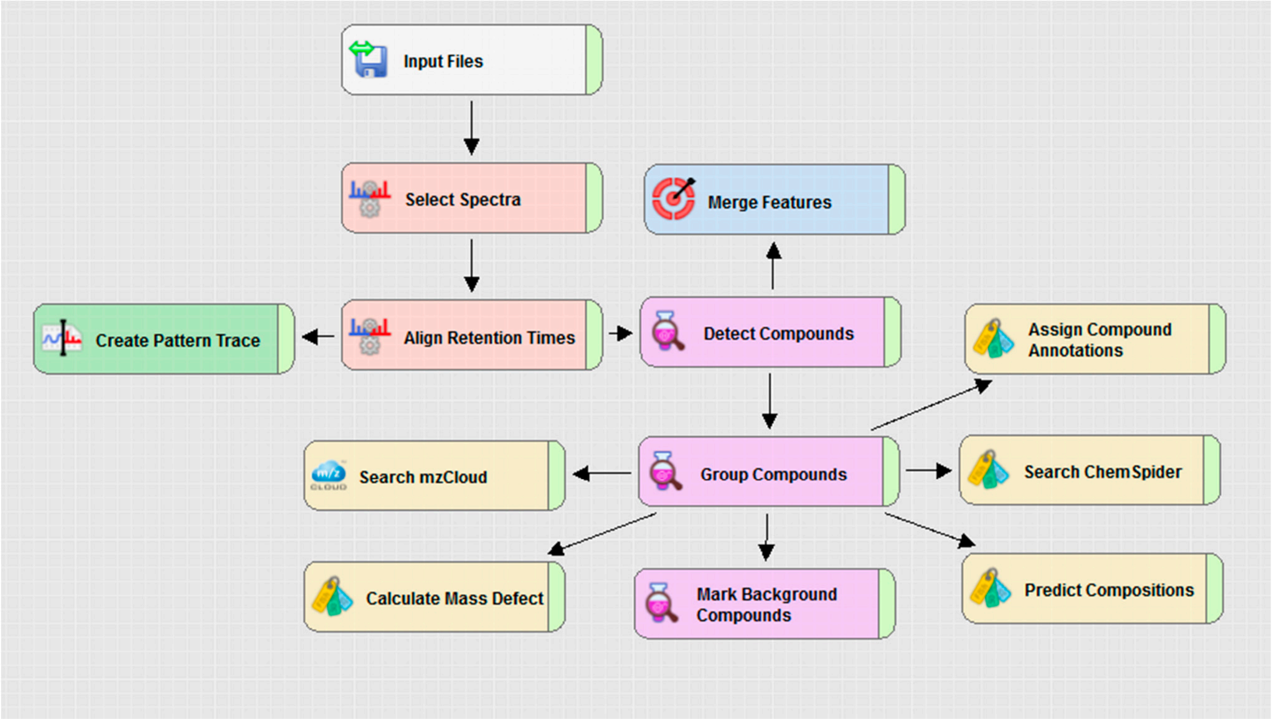

Figure S1. Workflow on Compound Discoverer used for flavonoid identification
